# Correlate tumor mutation burden with immune signatures in human cancers

**DOI:** 10.1186/s12865-018-0285-5

**Published:** 2019-01-11

**Authors:** Xiaosheng Wang, Mengyuan Li

**Affiliations:** 1 Biomedical Informatics Research Lab, School of Basic Medicine and Clinical Pharmacy, China Pharmaceutical University, Nanjing, 211198 China; 2 Cancer Genomics Research Center, School of Basic Medicine and Clinical Pharmacy, China Pharmaceutical University, Nanjing, 211198 China; 3 Big Data Research Institute, China Pharmaceutical University, Nanjing, 211198 China

**Keywords:** Tumor mutation burden, Immune signatures, Tumor immune microenvironment, Cancer immunotherapy, Cancer prognosis

## Abstract

**Background:**

Tumor mutation burden (TMB) has been associated with cancer immunotherapeutic response and cancer prognosis. Although many explorations have revealed that high TMB may yield many neoantigens to incite antitumor immune response, a systematic exploration of the correlation between TMB and immune signatures in different cancer types is lacking.

**Results:**

We classified cancer into the lower-TMB subtype and the higher-TMB subtype for each of 32 cancer types based on their somatic mutation data from the Cancer Genome Atlas (TCGA), and compared the expression levels of immune-related genes and gene-sets between both subtypes of cancers in each cancer type. In some cancer types most of the immune signatures analyzed were upregulated in the lower-TMB subtype, while in some other cancer types the immune signatures were prone to be upregulated in the higher-TMB subtype. However, the regulatory T cells, immune cell infiltrate, tumor-infiltrating lymphocytes, and cytokine signatures tended to be upregulated in the lower-TMB subtype, and the cancer-testis antigen (CTA) and pro-inflammatory signatures were inclined to be upregulated in the higher-TMB subtype. Importantly, high TMB was associated with elevated expression of *PD-L1* in diverse prevailing cancers. Furthermore, we found that higher TMB was associated with better survival prognosis in numerous cancer types while was associated with worse prognosis in a few cancer types.

**Conclusions:**

High TMB may inhibit immune cell infiltrations while promote CTAs expression and inflammatory response in cancer. In many common cancer types, higher TMB may respond favorably to anti-PD-1/PD-L1 immunotherapy. Our data implicate that higher-TMB patients could gain a more favorable prognosis in diverse cancer types if treated with immunotherapy, otherwise would have a poorer prognosis compared to lower-TMB patients.

**Electronic supplementary material:**

The online version of this article (10.1186/s12865-018-0285-5) contains supplementary material, which is available to authorized users.

## Background

Cancer immunotherapy is becoming increasingly noteworthy for its effectiveness in treating advanced and refractory cancers [1]. Particularly, the immune checkpoint blockade is being clinically used for treating diverse malignancies, such as melanoma [2, 3] and lung cancer [4]; the chimeric antigen receptor T cell therapy has been successfully utilized to treat refractory leukemia and lymphoma [5]. Nevertheless, these immunotherapies are beneficial to only 20% of cancer patients [6]. Thus, many efforts have been devoted to discovering the molecular determinants of immunotherapeutic responsiveness [7]. Some well-recognized molecular determinants include PD-L1 expression on tumor [8], DNA mismatch-repair deficiency [9], neoantigen load [10], and tumor-infiltrating lymphocytes [11]. Besides, to improve the efficacy of cancer immunotherapy, the combination of different immunotherapeutic methods [12, 13], or the combination of immunotherapy with other therapeutic approaches [14–17] have been explored.

A number of studies have explored the association between tumor mutation burden (TMB) and immunotherapy response [2–4, 18, 19]. These studies demonstrated that higher nonsynonymous mutation burden in tumors is inclined to form more neoantigens that make tumors to have higher immunogenicity, and thus result to improved clinical response to immunotherapy [4]. Nevertheless, a systematic exploration of the correlation between TMB and tumor immune activities in different cancer types remains lacking. To explore the association of TMB with tumor immunity in different cancer types, we compared the expression levels of immune-related genes and gene-sets between the lower-TMB subtype and the higher-TMB subtype of 32 cancer types based on the Cancer Genome Atlas (TCGA) data (https://portal.gdc.cancer.gov/). We tried to address several questions, including: Is the immune activity of the higher-TMB subtype different from that of the lower-TMB subtype of cancers? Are there any immune-related genes or gene-sets which are differentially expressed between the lower-TMB subtype and the higher-TMB subtype of cancers and whose expression is associated with clinical outcomes in cancer? Is the TMB itself associated with clinical outcomes in cancer?

## Results

### Association of TMB with regulatory T cell marker genes expression in human cancers

**Fig. 1:**
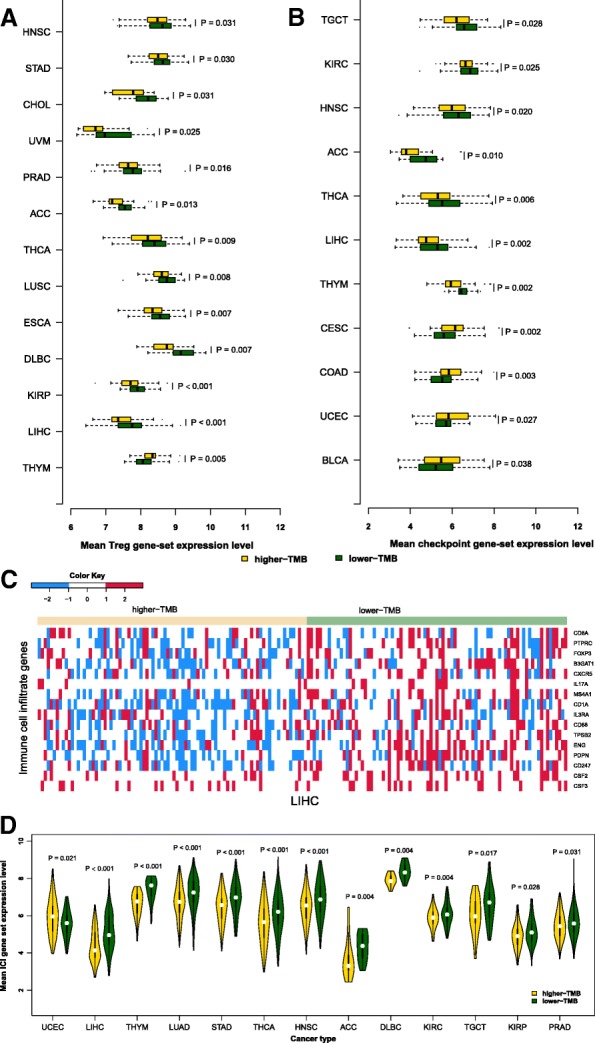
Comparison of the expression levels of Treg, immune checkpoint, and immune cell infiltrate gene signatures between the lower-TMB and the higher-TMB subtypes of cancers. **a** The cancer types in which the Treg gene-set is differentially expressed between the lower-TMB and the higher-TMB subtypes (Wilcoxon rank-sum test, *P* < 0.05). **b** The cancer types in which the immune checkpoint gene-set is differentially expressed between the lower-TMB and the higher-TMB subtypes (Wilcoxon rank-sum test, P < 0.05). **c** Heat-map for the expression levels of immune cell infiltrate genes in lower-TMB and higher-TMB LIHC. **d** The cancer types in which the immune cell infiltrate (ICI) gene-set is differentially expressed between the lower-TMB and the higher-TMB subtypes (Wilcoxon rank-sum test, *P*-value< 0.05)

Regulatory T (Treg) cells play an important role in the maintenance of tumor immunosuppression [20]. We compared expression levels of 70 tumor-infiltrating Treg gene signatures [21] between the lower-TMB and the higher-TMB cancers in each of the 32 cancer types. We found that seven genes (*ADPRH*, *IL1R1*, *KSR1*, *SOCS2*, *JAK1*, *NFAT5*, and *SSH1*) were more highly expressed in the lower-TMB subtype than in the higher-TMB subtype of more than 10 cancer types (Additional file 1: Table S1). Of note, *ADPRH* had significantly higher expression levels in the lower-TMB subtype of 14 cancer types. The expression levels of the Treg gene-set were significantly higher in the lower-TMB subtype of 12 cancer types (HNSC, STAD, CHOL, UVM, PRAD, ACC, THCA, LUSC, ESCA, DLBC, KIRP, and LIHC) while were significantly higher in the higher-TMB subtype of 1 cancer type (THYM) (Wilcoxon rank-sum test, *P* < 0.05) (Fig. 1a). Interestingly, 27 Treg genes were more highly expressed in lower-TMB LIHC versus 1 more highly expressed in higher-TMB LIHC (Fisher’s exact test, *P* = 1.2*10^− 8^, OR = 42.42). In contrast, 14 Treg genes were more highly expressed in lower-TMB THYM versus 28 more highly expressed in higher-TMB THYM (Fisher’s exact test, *P* = 0.016, OR = 0.38). These results suggest that the relatedness between TMB and Treg cells infiltration degree depends on cancer types, whereas the lower-TMB subtype is likely to have stronger Treg cells infiltration than the higher-TMB subtype in diverse cancers.

### Association of TMB with immune checkpoint genes expression in human cancers

Immune checkpoint molecules are important for tumor immune evasion [22]. We compared expression levels of 47 immune checkpoint genes [21] between the lower-TMB subtype and the higher-TMB subtype of cancers. We found that 12 genes had significantly higher expression levels in the higher-TMB subtype than in the lower-TMB subtype of at least 6 cancer types (Additional file 1: Table S2). The 12 genes included *LAG3*, *CD80*, *TNFSF9*, *IDO1*, *CD70*, *KIR3DL1*, *CTLA4*, *PD-1*, *PD-L1*, *PD-L2*, *TIGIT*, and *TNFRSF9*. Notably, *LAG3* had higher expression levels in the higher-TMB subtype of 10 cancer types versus 2 cancer types of which *LAG3* showed higher expression levels in the lower-TMB subtype. Interestingly, many immune checkpoint genes, which are established or promising targets for immune checkpoint blockade therapy, had significantly higher expression levels in the higher-TMB subtype of various cancers, such as *CTLA4*, *PD-1*, *PD-L1*, *PD-L2*, *LAG3*, *IDO1* and *TIGIT.* In contrast, 16 immune checkpoint genes (*C10orf54*, *CD200*, *CD40LG*, *ADORA2A*, *TNFSF14*, *BTLA*, *CD160*, *CD44*, *CD48*, *CD28*, *VTCN1*, *CD200R1*, *NRP1*, *TMIGD2*, *ICOS*, and *TNFSF15*) had significantly higher expression levels in the lower-TMB subtype than in the higher-TMB subtype of at least 6 cancer types.

The expression levels of the immune checkpoint gene-set were significantly higher in the lower-TMB subtype than in the higher-TMB subtype of TGCT, KIRC, HNSC, ACC, THCA, LIHC, and THYM, while were significantly higher in the higher-TMB subtype of CESC, COAD, UCEC, and BLCA (Wilcoxon rank-sum test, *P* < 0.05) (Fig. 1b). Of the 47 immune checkpoint genes, 22 were more highly expressed in higher-TMB CESC versus 3 more highly expressed in lower-TMB CESC (Fisher’s exact test, *P* = 1.3*10^− 5^, OR = 12.55). In addition, 20 immune checkpoint genes were more highly expressed in higher-TMB COAD versus 1 more highly expressed in lower-TMB COAD (Fisher’s exact test, *P* = 1.98*10^− 6^, OR = 33). In contrast, no any immune checkpoint gene was more highly expressed in higher-TMB LIHC versus 22 more highly expressed in lower-TMB LIHC (Fisher’s exact test, *P* = 1.88*10^− 8^), and the similar result was observed in THCA. These data indicate that the association between TMB and the immune checkpoint activity is cancer type dependent, with in some cancers TMB being positively correlated with the immune checkpoint activity while in some other cancers they showing an inverse correlation.

### Association of TMB with immune cell infiltration in human cancers

We compared the infiltration densities of 16 different immune cell subpopulations [23] between the lower-TMB subtype and the higher-TMB subtype of cancers. We found that 11 immune cell subpopulation marker genes had significantly higher expression levels in the lower-TMB subtype than in the higher-TMB subtype of at least 6 cancer types (Additional file 1: Table S3). The 11 genes included *ENG* (blood vessels), *CD45RO* (memory T cells), *CD20* (B cells), *CD1A* (immature Dendritic Cells (iDCs)), *TPSB2* (mast cells), *PDPN* (lymph vessels), *CXCR5* (follicular helper T (Tfh cells)), *IL3RA* (plasmacytoid Dendritic Cells (pDCs)), *CSF3* (neutrophils), *CD3* (T cells)*,* and *CD57* (activated T or NK cells). In contrast, only *CD68* (macrophages) had significantly higher expression levels in the higher-TMB subtype than in the lower-TMB subtype of at least 6 cancer types. It suggests that most of these immune cells have stronger infiltration in the lower-TMB subtype than in the higher-TMB subtype of cancers. Typically, 10 of the 16 immune cell subpopulation marker genes were more highly expressed in lower-TMB LIHC, but no one was more highly expressed in higher-TMB LIHC (Fig. 1c), indicating that heavy mutation load may inhibit immune cell infiltration in LIHC. Furthermore, we found 12 cancer types in which the expression levels of the immune cell subpopulation gene-set being significantly higher in the lower-TMB subtype, versus 1 cancer type in which the expression levels of this gene-set being significantly higher in the higher-TMB subtype (Fig. 1d). Again, this suggests that high TMB tends to inhibit immune cell infiltration in cancer.

### Association of TMB with tumor-infiltrating lymphocytes (TILs) infiltration in human cancers

**Fig. 2:**
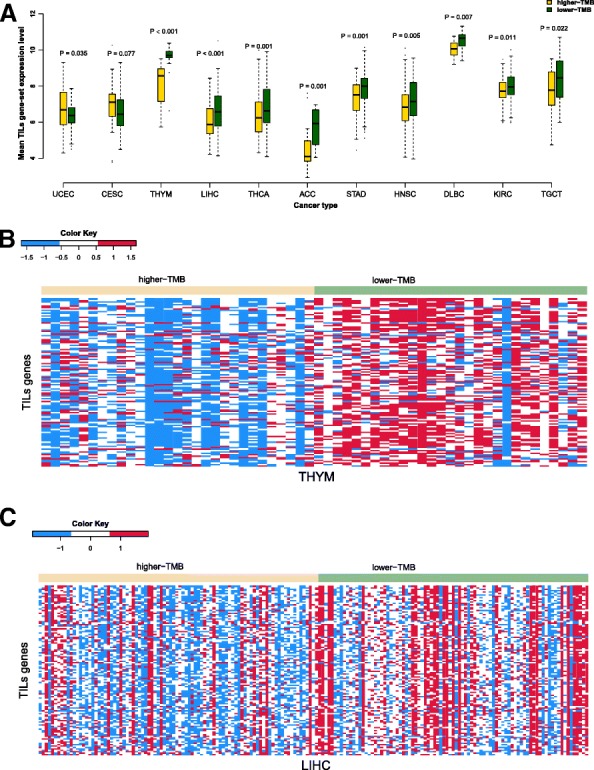
Comparison of the expression levels of tumor-infiltrating lymphocytes (TILs) genes between the lower-TMB and the higher-TMB subtypes of cancers. **a** The cancer types in which the TILs gene-set is differentially expressed between the lower-TMB and the higher-TMB subtypes (Wilcoxon rank-sum test, P-value< 0.05). **b** Heat-map for the expression levels of TILs genes in lower-TMB and higher-TMB THYM. **c** Heat-map for the expression levels of TILs genes in lower-TMB and higher-TMB LIHC

We compared expression levels of 120 TILs gene signatures [24] between the lower-TMB subtype and the higher-TMB subtype of cancers. We found that 90 genes had significantly higher expression levels in the lower-TMB subtype of at least 6 cancer types versus 5 having significantly higher expression levels in the higher-TMB subtype of at least 6 cancer types (Fisher’s exact test, *P* < 2.2*10^− 16^, OR = 67.11) (Additional file 1: Table S4). Notably, *GIMAP6* had significantly higher expression levels in the lower-TMB subtype than in the higher-TMB subtype of 15 cancer types. *GIMAP6* encodes the immunity-associated nucleotide 6 protein, a member of the GTPases of immunity-associated proteins family. This gene has been shown to be downregulated in several cancer types such as NSCLC [25] and LIHC [26]. Our results showed that this gene was more lowly expressed in higher-TMB LIHC than in lower-TMB LIHC. The expression levels of the TILs gene-set were significantly higher in the lower-TMB subtype of 9 cancer types (TGCT, KIRC, DLBC, HNSC, STAD, ACC, THCA, LIHC and THYM), while were significantly higher in the higher-TMB subtype of 2 cancer types (CESC and UCEC) (Fig. 2a). These results indicated that although the association between TMB and TILs infiltration was cancer type dependent, high TMB tended to inhibit TILs infiltration in various cancer types. Strikingly, 99 of the 120 TILs genes were more highly expressed in lower-TMB THYM compared to zero showing higher expression levels in higher-TMB THYM (Fisher’s exact test, *P* < 2.2*10^− 16^) (Fig. 2b). In addition, 88 TILs genes were more highly expressed in lower-TMB LIHC versus 3 more highly expressed in higher-TMB LIHC (Fisher’s exact test, P < 2.2*10^− 16^, OR = 104.46) (Fig. 2c).

### Association of TMB with cancer-testis antigen genes expression in human cancers

Cancer-testis antigens (CTAs) are a group of immunogenic proteins that are aberrantly activated in a variety of cancer types, and thus are important targets for developing cancer immunotherapy [27]. We compared expression levels of 276 CTA genes [28] between the lower-TMB subtype and the higher-TMB subtype of cancers. We found that 28 CTA genes had significantly higher expression levels in the lower-TMB subtype of at least 6 cancer types, while 63 had significantly higher expression levels in the higher-TMB subtype of at least 6 cancer types (Fisher’s exact test, *P* = 8.23*10^− 5^, OR = 0.38) (Additional file 1: Table S5). Of note, 6 CTA genes *CEP55*, *KIF2C*, *TTK*, *OIP5*, *CASC5*, and *NUF2* had higher expression levels in the higher-TMB subtype of at least 15 cancer types, while had higher expression levels in the lower-TMB subtype of at most 3 cancer types. Interestingly, a number of genes encoding CTAs that are potentially useful for developing cancer vaccines were in the list of 63 CTA genes with higher expression levels in the higher-TMB subtype, such as *MAGEA* (*MAGEA*-*1*, *2*, *3*, *4*, *6*, *8*, *9B*, *10*, *11*, *12*), *NY-ESO-1*, and *PRAME*.

**Fig. 3:**
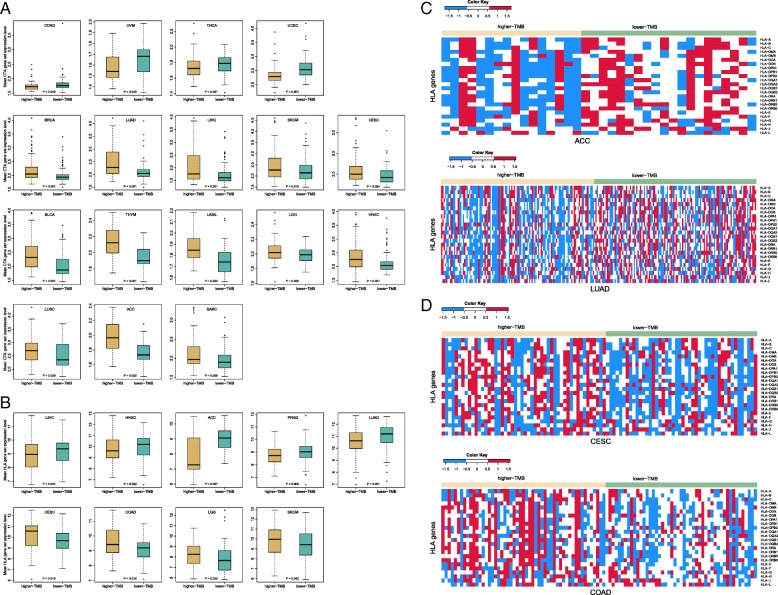
Comparison of the expression levels of cancer-testis antigen (CTA) and HLA genes between the lower-TMB and the higher-TMB subtypes of cancers. **a** The cancer types in which the CTA gene-set is differentially expressed between the lower-TMB and the higher-TMB subtypes (Wilcoxon rank-sum test, P-value< 0.05). **b** The cancer types in which the HLA gene-set is differentially expressed between the lower-TMB and the higher-TMB subtypes (Wilcoxon rank-sum test, P-value< 0.05). **c** Heat-maps for the expression levels of HLA genes in lower-TMB and higher-TMB ACC and LUAD. **d** Heat-maps for the expression levels of HLA genes in lower-TMB and higher-TMB CESC and COAD

The expression levels of the CTA gene-set were significantly higher in the higher-TMB subtype of 13 cancer types, including BRCA, LUAD, LIHC, SKCM, CESC, BLCA, THYM, LAML, LGG, HNSC, LUSC, ACC, and SARC, and were significantly higher in the lower-TMB subtype of 4 cancer types (COAD, UVM, THCA, and UCEC) (Fig. 3a). In many cancer types, the number of CTA genes with higher expression levels in the higher-TMB subtype far exceeded that of CTA genes with higher expression levels in the lower-TMB subtype. For example, 111 CTA genes were more highly expressed in higher-TMB LUAD versus 20 more highly expressed in lower-TMB LUAD (Fisher’s exact test, *P* < 2.2*10^− 16^, OR = 8.58); 102 CTA genes were more highly expressed in higher-TMB BRCA versus 29 more highly expressed in lower-TMB BRCA (Fisher’s exact test, *P* = 1.83*10^− 13^, OR = 4.98). These results suggest that high TMB is associated with elevated expression of many CTAs in cancer.

### Association of TMB with HLA genes expression in human cancers

HLA (human leukocyte antigen) plays an important role in regulating the immune system in humans [29]. We compared expression levels of 24 HLA genes (with expression values available in the TCGA RNA-Seq data) between the lower-TMB subtype and the higher-TMB subtype of cancers. We found that 6 genes (*HLA-J*, *DOA*, *DOB*, *DPB1*, *DQA1*, and *DQB2*) had significantly higher expression levels in the lower-TMB subtype of at least 5 cancer types, while no any HLA gene showed significantly higher expression levels in the higher-TMB subtype of at least 5 cancer types (Additional file 1: Table S6). The expression levels of the HLA gene-set were significantly higher in the lower-TMB subtype of LIHC, HNSC, ACC, PRAD, and LUAD, and were significantly higher in the higher-TMB subtype of CESC, COAD, LGG, and SKCM (Fig. 3b). In LIHC, HNSC, ACC, PRAD, and LUAD, there were much more HLA genes showing higher expression levels in the lower-TMB subtype than those showing higher expression levels in the higher-TMB subtype (10 versus 1, 5 versus 0, 14 versus 0, 10 versus 0, and 16 versus 0 for LIHC, HNSC, ACC, PRAD, and LUAD, respectively) (Fig. 3c). In contrast, in CESC, COAD, and SKCM, there were much more HLA genes with higher expression levels in the higher-TMB subtype than those with higher expression levels in the lower-TMB subtype (12 versus 0, 12 versus 0, and 6 versus 0 for CESC, COAD, and SKCM, respectively) (Fig. 3d). These results suggest that the association between TMB and HLA expression is cancer type dependent.

### Association of TMB with cytokine-related genes expression in human cancers

Cytokines are important components of the tumor immune microenvironment (TIM) [30]. Of 261 cytokine and cytokine receptor (CCR) genes [31], 93 showed significantly higher expression levels in the lower-TMB subtype versus 31 showing significantly higher expression levels in the higher-TMB subtype of at least 6 cancer types (Fisher’s exact test, *P* = 1.77*10^− 10^, OR = 4.10) (Additional file 1: Table S7). Notably, *TNFAIP8L3*, *CCL14*, *CX3CR1*, *CCL21*, *IL1R1*, and *IL33* had higher expression levels in the lower-TMB subtype of at least 13 cancer types, while had higher expression levels in the higher-TMB subtype of at most 2 cancer types. In contrast, *ILF2* showed higher expression levels in the higher-TMB subtype of 13 cancer types, while showed higher expression levels in the lower-TMB subtype of 1 cancer type.

**Fig. 4:**
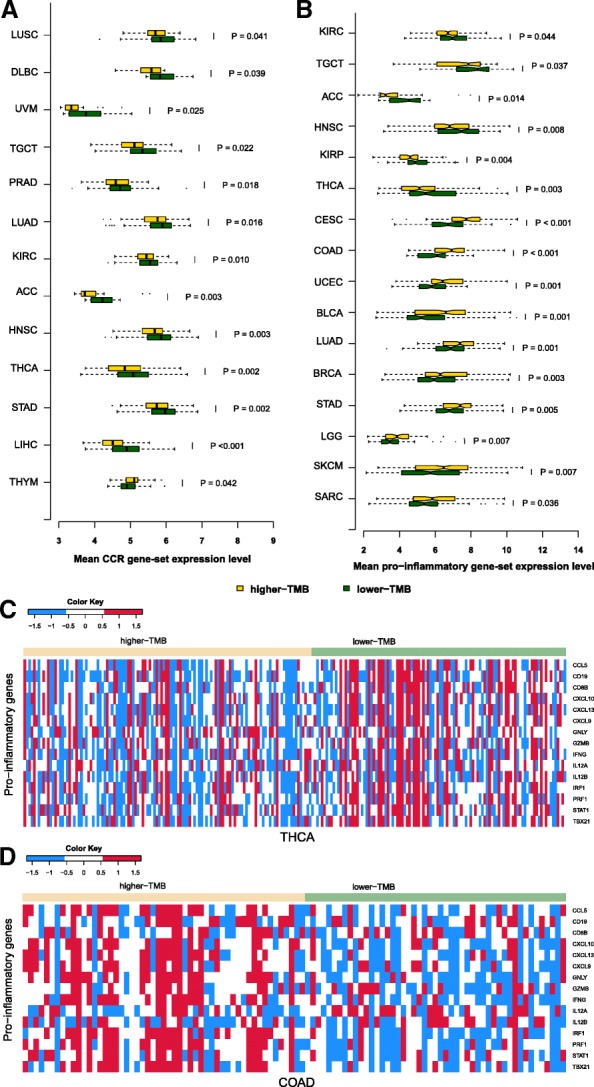
Comparison of the expression levels of cytokine-related and pro-inflammatory genes between the lower-TMB and the higher-TMB subtypes of cancers. **a** The cancer types in which the cytokine and cytokine receptor (CCR) gene-set is differentially expressed between the lower-TMB and the higher-TMB subtypes (Wilcoxon rank-sum test, P-value< 0.05). **b** The cancer types in which the pro-inflammatory gene-set is differentially expressed between the lower-TMB and the higher-TMB subtypes (Wilcoxon rank-sum test, P-value< 0.05). **c** Heat-map for the expression levels of pro-inflammatory genes in lower-TMB and higher-TMB THCA. **d** Heat-map for the expression levels of pro-inflammatory genes in lower-TMB and higher-TMB COAD

Interestingly, the expression levels of the CCR gene-set were significantly higher in the lower-TMB subtype of 12 cancer types (LUSC, DLBC, UVM, TGCT, PRAD, LUAD, KIRC, ACC, HNSC, THCA, STAD, and LIHC), while were significantly higher in the higher-TMB subtype of THYM solely (Fig. 4a). It suggests that high TMB may lead to depressed cytokine activity in diverse cancers. Indeed, 98 CCR genes were more highly expressed in lower-TMB LIHC versus 7 more highly expressed in higher-TMB LIHC (Fisher’s exact test, *P* < 2.2*10^− 16^, OR = 21.71). In addition, 86 CCR genes were more highly expressed in lower-TMB THCA versus 2 more highly expressed in higher-TMB THCA (Fisher’s exact test, P < 2.2*10^− 16^, OR = 63.35).

### Association of TMB with pro-inflammatory genes expression in human cancers

Inflammatory responses play important roles in regulating the TIM [32]. We compared expression levels of 15 pro-inflammatory genes [33] between the lower-TMB subtype and the higher-TMB subtype of cancers. We found that 10 pro-inflammatory genes were more highly expressed in the lower-TMB subtype versus 4 more highly expressed in the higher-TMB subtype of at least 5 cancer types (Additional file 1: Table S8). Notably, *CXCL9*, *CXCL10*, and *IFNG* had significantly higher expression levels in the higher-TMB subtype of at least 10 cancer types, while had significantly higher expression levels in the lower-TMB subtype of at most 4 cancer types. The expression levels of the pro-inflammatory gene-set were significantly higher in the lower-TMB subtype of 6 cancer types (KIRC, TGCT, ACC, HNSC, KIRP, and THCA), and were significantly higher in the higher-TMB subtype of 10 cancer types (CESC, COAD, UCEC, BLCA, LUAD, BRCA, STAD, LGG, SKCM, and SARC) (Fig. 4b). Interestingly, 12 of the 15 pro-inflammatory genes were more highly expressed in lower-TMB THCA versus zero more highly expressed in higher-TMB THCA (Fig. 4c). In contrast, 11 pro-inflammatory genes were more highly expressed in higher-TMB COAD versus zero more highly expressed in lower-TMB COAD (Fig. 4d). These results indicate that the association between TMB and pro-inflammatory activity is cancer type dependent, with in some cancer types high TMB enhancing pro-inflammatory activity while in some other cancer types high TMB inhibiting pro-inflammatory activity in cancer.

### Associations among TMB, immune signatures, and cancer prognosis

**Fig. 5:**
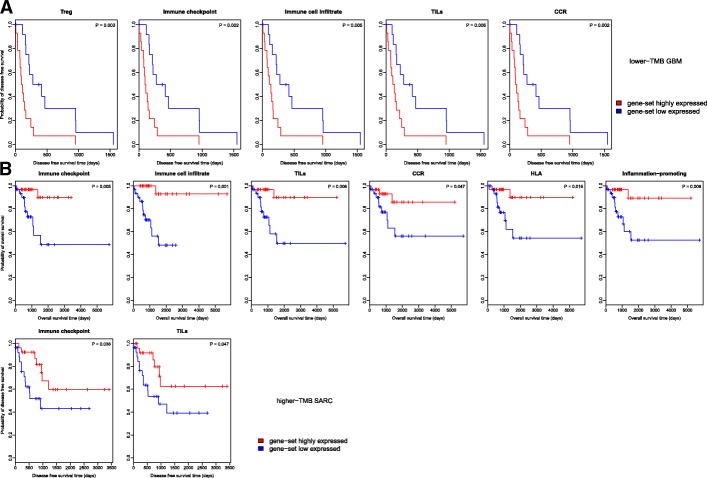
Association among TMB, immune signatures, and cancer prognosis. **a** Kaplan-Meier survival curves show that the elevated expression of Treg, immune checkpoint, immune cell infiltrate, TILs, and CCR gene-sets are consistently associated with worse disease free survival (DFS) prognosis in lower-TMB GBM (log-rank test, P < 0.05). **b** The elevated expression of immune checkpoint, TILs, HLA, CCR, and pro-inflammatory gene-sets are consistently associated with better overall survival (OS) and/or DFS prognosis in higher-TMB SARC (log-rank test, P < 0.05)

To explore the association among TMB, immune signatures, and cancer prognosis, we compared survival prognosis (overall survival (OS) and disease free survival (DFS)) between immune gene-set higher-expression-level and lower-expression-level lower-TMB cancers and higher-TMB cancers, respectively. We found that some cancer types showed a significant correlation between immune gene-set expression and survival prognosis in the lower-TMB subtype but not in the higher-TMB subtype. For example, higher expression levels of the Treg, immune checkpoint, immune cell infiltrate, TILs, and CCR gene-sets were consistently associated with worse DFS in lower-TMB GBM (log-rank test, *P* < 0.05), but there was no any immune gene-set whose expression was associated with survival prognosis in higher-TMB GBM (Fig. 5a). In contrast, some cancer types showed a significant correlation between immune gene-set expression and survival prognosis in the higher-TMB subtype but not in the lower-TMB subtype. For example, higher expression levels of the immune checkpoint, immune cell infiltrate, TILs, CCR, HLA, and pro-inflammatory gene-sets were consistently associated with better OS and/or DFS in higher-TMB SARC, but there was no any immune gene-set whose expression was associated with survival prognosis in lower-TMB SARC (Fig. 5b). These results indicate that there exist significant associations among TMB, immune signatures, and survival prognosis in some cancer types.

## Discussion

**Fig. 6:**
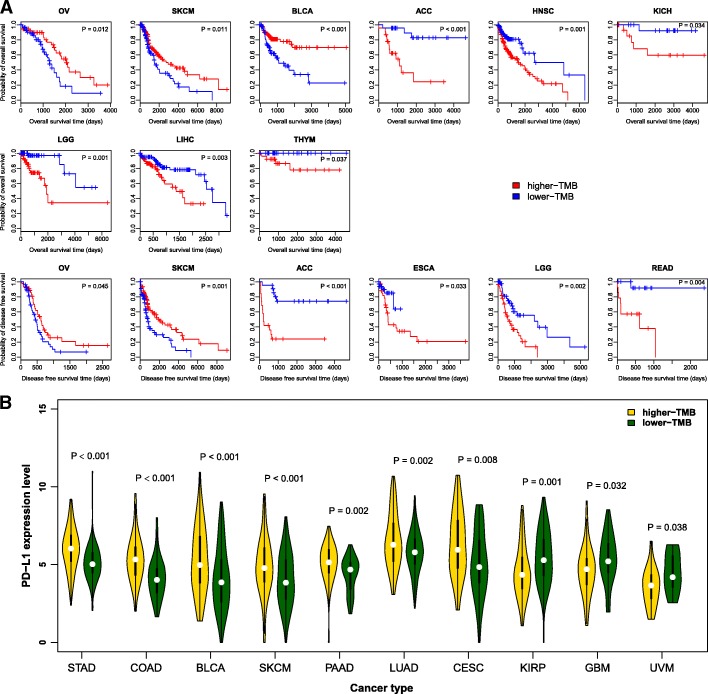
Association of TMB with survival prognosis and *PD-L1* expression in cancers. **a** Kaplan-Meier survival curves show that TMB is associated with survival prognosis in diverse cancer types (log-rank test, P < 0.05). **b** *PD-L1* is differentially expressed between the lower-TMB and the higher-TMB subtypes of diverse cancer types (Student’s *t* test, P < 0.05, fold change > 1.5)

We analyzed the associations between TMB and diverse immune signatures in 32 human cancer types. We found that these associations were generally cancer type dependent. For example, most of the immune signatures were upregulated in the lower-TMB subtype of HNSC, ACC, THCA, and LIHC, while were downregulated in the lower-TMB subtype of CESC relative to their higher-TMB subtypes. However, the Treg cells, immune cell infiltrate, TILs, and CCR signatures were inclined to be upregulated in the lower-TMB subtype of various cancer types, suggesting that high TMB may inhibit immune cell infiltration in the TIM. In contrast, the CTA and pro-inflammatory signatures tended to be upregulated in the higher-TMB subtype of various cancer types, suggesting that high TMB may promote CTA expression and tumor inflammatory response. Interestingly, HNSC, ACC, THCA, and LIHC were the cancer types in which TMB and the immune gene-set expression alteration had a significant correlation for almost all the immune signatures analyzed (Table 1). It implies that TMB could have a significant impact on the TIM in these cancer types. In fact, when we compared survival prognosis between the lower-TMB subtype and the higher-TMB subtype of cancers, we found that the lower-TMB subtype had better OS and/or DFS prognosis than the higher-TMB subtype in three of the four cancer types including HNSC, ACC, and LIHC (Fig. 6a). These data suggest that TMB is associated with survival prognosis in some cancer types, and that the mechanism underlying this association could lie in the marked differences in immune cell infiltration densities and immune activities between the lower-TMB subtype and the higher-TMB subtype of these cancers.

**Table 1:** Comparison of immune activities between the lower-TMB and the higher-TMB subtypes of cancers

Immune signatures	Genes upregulated in the lower-TMB subtype of various cancer types ^a^	Genes upregulated in the higher-TMB subtype of various cancer types ^b^	Cancer types in which the immune signature is upregulated in the lower-TMB subtype	Cancer types in which the immune signature is upregulated in the higher-TMB subtype
Treg	*ADPRH*, *IL1R1*, *KSR1*, *SOCS2*, *JAK1*, *NFAT5*, *SSH1*	*TFRC*, *ETV7*, *ADAT2*, *PD-L1*, *IL12RB2*	HNSC, STAD, CHOL, UVM, PRAD, ACC, THCA, LUSC, ESCA, DLBC, KIRP, LIHC	THYM
immune checkpoint	*C10orf54*, *CD200*, *CD40LG*, *ADORA2A*, *TNFSF14*, *BTLA*, *CD160*, *CD44*, *CD48*, *CD28*, *VTCN1*, *CD200R1*, *NRP1*, *TMIGD2*, *ICOS*, *TNFSF15*	*LAG3*, *CD80*, *TNFSF9*, *IDO1*, *CD70*, *KIR3DL1*, *CTLA4*, *PD-1*, *PD-L1*, *PD-L2*, *TIGIT*, *TNFRSF9*	TGCT, KIRC, HNSC, ACC, THCA, LIHC, THYM	CESC, COAD, UCEC, BLCA
immune cell infiltrate	*ENG*, *CD45RO*, *CD20*, *CD1A*, *TPSB2*, *PDPN*, *CXCR5*, *IL3RA*, *CSF3*, *CD3*, *CD57*	*CD68*	PRAD, KIRP, TGCT, KIRC, DLBC, ACC, HNSC, THCA, STAD, LUAD, THYM, LIHC	UCEC
TILs	*GIMAP6*, *CFH*, *ITGA4*, *FAM65B*, *GVIN1*, *ARHGAP15*, *ARHGAP25*, *GIMAP4*, *GIMAP7*, *GPSM3*, *IL16*, *PIK3CD*, *PRKCB*, *SELL*, *GIMAP5*, *INPP5D*, *NLRC3*, *PRKCQ*, *TRAT1*	*XCL1*, *SPNS1*, *VAMP5*, *TIGIT*	TGCT, KIRC, DLBC, HNSC, STAD, ACC, THCA, LIHC, THYM	CESC, UCEC, COAD
CTA	*CEP55*, *KIF2C*, *TTK*, *OIP5*, *CASC5*, *NUF2*, *MAGEA*, *MAGEB*, *MAGEC*, *PAGE*, *NY-ESO-1*, *PRAME*	*RGS22*, *TDRD6*, *TMEM108*	COAD, UVM, THCA, UCEC	BRCA, LUAD, BLCA, HNSC, THYM, LIHC, LAML, ACC, SARC, SKCM, CESC, LGG, LUSC
HLA	*HLA-J*, *DOA*, *DOB*, *DPB1*, *DQA1*, *DQB2*	NA	LIHC, HNSC, ACC, PRAD, LUAD	CESC, COAD, LGG, SKCM
CCR	*TNFAIP8L3*, *CCL14*, *CX3CR1*, *CCL21*, *IL1R1*, *IL33*, *CCL19*, *CCR6*, *IL16*, *IL17D*, *TGFB2*, *TGFBR2*, *BMP3*, *CXCL12*, *TNFSF8*	*ILF2*, *CXCL9*, *CXCL10*, *CXCL11*, *ILKAP*, *IFNG*	LUSC, DLBC, UVM, TGCT, PRAD, LUAD, KIRC, ACC, HNSC, THCA, STAD, LIHC	THYM
pro-inflammatory	*IL12A*, *IL12B*, *PRF1*, *TBX21*	*CXCL9*, *CXCL10*, *IFNG*, *GZMB*, *CXCL13*, *STAT1*, *IRF1*, *GNLY*	KIRC, TGCT, ACC, HNSC, KIRP, THCA	CESC, COAD, UCEC, BLCA, LUAD, BRCA, STAD, LGG, SKCM, SARC

^a^Genes with higher expression levels in the lower-TMB subtype than in the higher-TMB subtype of multiple cancer types (only some representative genes are listed)

^b^Genes with higher expression levels in the higher-TMB subtype than in the lower-TMB subtype of multiple cancer types (only some representative genes are listed)

**Table 2:** 32 TCGA cancer types used in this study

Cancer	Full name	# cancer samples ^c^	# normal samples	# higher-TMB samples	# lower-TMB samples
ACC	adrenocortical carcinoma	79	0	16	20
BLCA	bladder urothelial carcinoma	408	19	99	97
BRCA	breast invasive carcinoma	1100	112	241	245
CESC	cervical squamous-cell carcinoma and endocervical adeno-carcinoma	306	3	50	46
CHOL	cholangiocarcinoma	36	9	9	9
COAD	colon adenocarcinoma	287	41	53	49
DLBC	lymphoid neoplasm diffuse large B-cell lymphoma	48	0	12	12
ESCA	esophageal carcinoma	185	11	45	46
GBM	glioblastoma multiforme	166	5	41	30
HNSC	head and neck squamous cell carcinoma	522	44	127	123
KICH	kidney chromophobe	66	25	17	17
KIRC	kidney renal clear cell carcinoma	534	72	120	110
KIRP	kidney renal papillary cell carcinoma	291	32	70	68
LAML	acute myeloid leukemia	173	0	24	24
LGG	brain lower-grade glioma	530	0	69	66
LIHC	liver hepatocellular carcinoma	373	50	90	87
LUAD	lung adenocarcinoma	517	59	120	127
LUSC	lung squamous cell carcinoma	501	51	45	45
OV	ovarian serous cystadeno-carcinoma	307	0	29	22
PAAD	pancreatic adeno-carcinoma	179	4	46	38
PCPG	pheochromocytoma and paraganglioma	184	3	45	41
PRAD	prostate adenocarcinoma	498	52	119	113
READ	rectum adenocarcinoma	95	10	19	20
SARC	sarcoma	263	2	63	64
SKCM	skincutaneous melanoma	472	1	117	118
STAD	stomach adenocarcinoma	415	35	90	85
TGCT	testicular germ-cell tumors	156	0	39	35
THCA	thyroid carcinoma	509	59	122	108
THYM	thymoma	120	2	29	29
UCEC	uterine corpus endometrial carcinoma	370	11	59	60
UCS	uterine carcino-sarcoma	57	0	14	13
UVM	uveal melanoma	80	0	20	19

^c^The numbers of cancer samples with both somatic mutations and gene expression profiles data

Interestingly, we found that high TMB was associated with elevated pro-inflammatory immune activity while depressed immune cell infiltration in diverse cancers. These findings appear to be contradictory and disagree with the established notion that high TMB may yield numerous neoantigens that incite anti-tumor immune response [4]. The possible explanations are that high TMB is often associated with genome instability that may inhibit anti-tumor immune response [34], and that the increased pro-inflammatory immune activity could be attributed to the higher percent of tumor necrosis component elicited by gene mutations in the higher-TMB cancer.

A recent study explored the landscape of TMB across 100,000 cancer cases in over 100 tumor types, and revealed that there were a substantial number of high-TMB cases across nearly every cancer type [35]. It justifies that the comparisons of high-TMB with low-TMB cases within each cancer type in the current study are sensible, although the methods in measuring the high-TMB and low-TMB are distinct between both studies. In another recent study [19], Goodman et al. revealed that higher TMB was associated with better clinical outcome in diverse cancers treated with various immunotherapies. However, our analyses showed that higher TMB was associated with better survival (OS and/or DFS) prognosis in SKCM, OV, and BLCA, while was associated with worse prognosis in ACC, ESCA, THYM, LIHC, LGG, HNSC, KICH, and READ based on the TCGA data (Fig. 6a). Seemingly, the revealed associations between TMB and cancer prognosis exhibit discrepancy between both studies. The main reason behind the discrepancy could be that most of the TCGA patients were not treated with immunotherapy. Indeed, for the TCGA cases likely with immunotherapy such as melanoma, higher TMB was associated with better prognosis, consistent with the conclusion drawn in [19]. Our data, together with the data from [19], implicate that higher-TMB patients could gain a more favorable prognosis compared to lower-TMB patients in diverse cancers if treated with immunotherapy, otherwise higher-TMB patients would have an unfavorable prognosis compared to lower-TMB patients.

Previous studies have shown that PD-L1 expression is positively associated with response to anti-PD-L1 immunotherapy [36]. Our data showed that *PD-L1* had significantly higher expression levels in the higher-TMB subtype than in the lower-TMB subtype of 7 cancer types (STAD, COAD, BLCA, SKCM, PAAD, LUAD, and CESC), compared to 3 cancer types (KIRP, GBM, and UVM) of which *PD-L1* was more lowly expressed in the higher-TMB subtype (Student’s *t* test, *P* < 0.05, fold change > 1.5) (Fig. 6b). It indicates that in the diverse prevailing malignancies, higher TMB could respond favorably to anti-PD-1/PD-L1 immunotherapy, bolstering the conclusion drawn in [19].

**Fig. 7:**
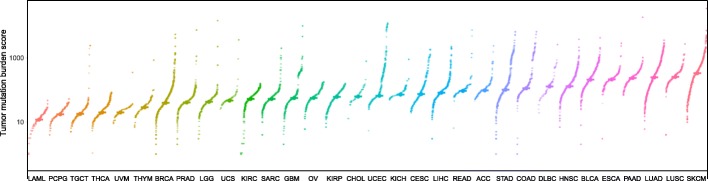
Distribution of TMB scores across 32 cancer types

In this study, we defined the higher-TMB and the lower-TMB subtypes of cancers on the basis of the TMB scores in the individual cancer types instead of across all cancer types that was a different approach from prior studies [4, 19, 35]. We used this approach mainly considering the intertumor heterogeneity. Indeed, there were marked gaps in TMB among different cancer types, with some cancers having high TMB such as SKCM, LUSC, LUAD, PAAD, ESCA, and BLCA while some other cancers having low TMB such as LAML, PCPG, TGCT, THCA, and UVM in general (Fig. 7). If we defined the higher-TMB and the lower-TMB subtypes of cancers based on the TMB scores across all cancer types, the sample size of the lower-TMB subtype in the highly-mutated cancer types, and the sample size of the higher-TMB subtype in the lowly-mutated cancer types would be too small to perform effective comparisons in these cancer types. As a result, it would be difficult to look into the correlations between TMB and immune activities in these cancer types. Interestingly, a recent pan-cancer analysis showed that the anti-tumor immune response could be more effective when the immune system robustly responded against a few antigens other than diversely responded against numerous different antigens [37]. It implicates that even in some cancer types with low TMB, anti-tumor immune response may take effect if the immune system heavily targets a few proper neoantigens.

There are several limitations in the present study. First, we have used a relatively loose significance level (*P* < 0.05) to identify differentially expressed genes (or gene-sets) when tens or hundreds of genes were tested. We did not use the method of adjusting for multiple tests such as the false discovery rate (FDR) [38] to define a more stringent significance threshold considering that the numbers of multiple tests were less than 100 in most of the cases, and were only eight in the comparisons of gene-set expression. Second, the cutoff for defining higher-TMB samples (the samples with TMB scores of upper quartile) and lower-TMB samples (the samples with TMB scores of lower quartile) was somewhat arbitrary. Nevertheless, when we reanalyzed the data using two different cutoffs to define higher-TMB samples versus lower-TMB samples (upper third vs. lower third, and upper half vs. lower half, respectively), we obtained the consistent results (Additional file 2: Table S9).

## Conclusions

High TMB may inhibit immune cell infiltrations while promote CTAs expression and inflammatory response in cancer. High TMB was associated with elevated expression of *PD-L1* in diverse prevailing cancers. Higher TMB was associated with better clinical outcomes in SKCM, OV, and BLCA, while was associated with worse prognosis in ACC, ESCA, THYM, LIHC, LGG, HNSC, KICH, and READ. Our data provide insights into the correlation between TMB and tumor immune response in different types of cancers, and have potential clinical implications for cancer immunotherapy.

## Methods

### Comparisons of expression levels of genes and gene-sets between two classes of samples

The TCGA RNA-Seq gene expression data (Level 3) were normalized by base-2 log transformation. Student’s *t* test was used to compare expression levels of a single gene between two groups of samples, and Wilcoxon rank-sum test was used to compare expression levels of a gene-set between two groups of samples.

### Definition of the higher-TMB and the lower-TMB subtypes of cancers

Consistent with a method we proposed previously [39], the TMB score of a tumor sample was calculated as follows:

total number of truncating mutations * 2.0 + total number of non-truncating mutations * 1.0

Nonsense, frame-shift deletion or insertion, and splice-site mutations were included in the truncating mutation category, and missense, in-frame deletion or insertion, and nonstop mutations were included in the non-truncating mutation category. The higher-TMB subtype (samples with TMB scores higher than the third quartile value) and the lower-TMB subtype (samples with TMB scores lower than the first quartile value) were defined in each individual cancer type based on the TMB scores of its tumor samples.

### Survival analyses

Besides the higher-TMB and the lower-TMB subtypes, we also defined two classes of cancer patients based on gene-set expression levels that indicate the activity of immune signatures. The expression level of a gene-set was evaluated as the average expression value of all the genes in the gene-set. The Kaplan-Meier method was used to compare survival (OS and DFS) between two classes of cancer patients (higher-TMB versus lower-TMB, and gene-set higher-expression-level (upper half) versus gene-set lower-expression-level (bottom half)). The log-rank test was used to evaluate the significance of survival-time differences between two classes of cancer patients.

### Statistical and computational analyses

For all statistical tests, a two-tailed *P* < 0.05 was considered statistically significant. All the statistical and computational analyses were performed using R programming (version 3.2.3, https://www.r-project.org/).

## Additional files

Additional file 1: **Table S1.** Comparison of regulatory T cell marker genes expression levels between the lower-TMB and the higher-TMB subtypes of cancers. **Table S2.** Comparison of immune checkpoint genes expression levels between the lower-TMB and the higher-TMB subtypes of cancers. **Table S3.** Comparison of immune cell infiltrate genes expression levels between the lower-TMB and the higher-TMB subtypes of cancers. **Table S4.** Comparison of tumor-infiltrating lymphocytes genes expression levels between the lower-TMB and the higher-TMB subtypes of cancers. **Table S5.** Comparison of cancer-testis antigen genes expression levels between the lower-TMB and the higher-TMB subtypes of cancers. **Table S6.** Comparison of HLA genes expression levels between the lower-TMB and the higher-TMB subtypes of cancers. **Table S7.** Comparison of cytokine and cytokine receptor genes expression levels between the lower-TMB and the higher-TMB subtypes of cancers. **Table S8.** Comparison of pro-inflammation genes expression levels between the lower-TMB and the higher-TMB subtypes of cancers. (XLSX 130 kb)

Additional file 2: **Table S9.** Comparison of the results obtained by using different cutoffs to define the higher-TMB and the lower-TMB tumor samples. (XLSX 21 kb)

## Abbreviations

ACC: Adrenocortical carcinoma
BLCA: Bladder urothelial carcinoma
BRCA: Breast invasive carcinoma
CCR: Cytokine and cytokine receptor
CESC: Cervical squamous-cell carcinoma and endocervicaladeno-carcinoma
CHOL: Cholangiocarcinoma
COAD: Colon adenocarcinoma
CTA: Cancer-testis antigen
CTLA4: Cytotoxic T-lymphocyte-associated protein 4
DFS: Disease free survival
DLBC: Lymphoid neoplasm diffuse large B-cell lymphoma
ESCA: Esophageal carcinoma
FDR: False discovery rate
GBM: Glioblastoma multiforme
HLA: Human leukocyte antigen
HNSC: Head and neck squamous cell carcinoma
iDCs: Immature Dendritic Cells
KICH: Kidney chromophobe
KIRC: Kidney renal clear cell carcinoma
KIRP: Kidney renal papillary cell carcinoma
LAML: Acute myeloid leukemia
LGG: Brain lower-grade glioma
LIHC: Liver hepatocellular carcinoma
LUAD: Lung adenocarcinoma
LUSC: Lung squamous cell carcinoma
NSCLC: Non-small cell lung cancer
OR: Odds ratio
OS: Overall survival
OV: Ovarian serous cystadenocarcinoma
PAAD: Pancreatic adeno-carcinoma
PD-1: Programmed cell death protein 1
pDCs: Plasmacytoid Dendritic Cells
PD-L1: Programmed cell death 1 ligand
PRAD: Prostate adenocarcinoma
READ: Rectum adenocarcinoma
SARC: Sarcoma
SKCM: Skincutaneous melanoma
STAD: Stomach adenocarcinoma
TCGA: The Cancer Genome Atlas
Tfh: Follicular helper T
TGCT: Testicular germ-cell tumors
THCA: Thyroid carcinoma
THYM: Thymoma
TILs: Tumor-infiltrating lymphocytes
TIM: Tumor immune microenvironment
TMB: Tumor mutation burden
Treg: Regulatory T
UCEC: Uterine corpus endometrial carcinoma
UCS: Uterine carcino-sarcoma
UVM: Uveal melanoma
